# Identification of TC2N as a novel promising suppressor of PI3K-AKT signaling in breast cancer

**DOI:** 10.1038/s41419-019-1663-5

**Published:** 2019-05-29

**Authors:** Xiang-lin Hao, Li-yun Gao, Xiao-juan Deng, Fei Han, Hong-qiang Chen, Xiao Jiang, Wen-bin Liu, Dan-dan Wang, Jian-ping Chen, Zhi-hong Cui, Lin Ao, Jia Cao, Jin-yi Liu

**Affiliations:** 1 Institute of Toxicology, College of Preventive Medicine, Third Military Medical University, Chongqing, 400038 PR China; 2 School of Public Health, Xinxiang Medical University, Xinxiang, PR China; 3 Cooperative innovation center of molecular diagnosis and medical inspection technology, Beijing, PR China

**Keywords:** Breast cancer, Phosphorylation

## Abstract

Although TC2N has proven to be an oncogene in lung cancer, its biological function and molecular mechanisms in other cancer still remains unclear. Here, we investigate in breast cancer that TC2N expression is sharply overexpressed in breast cancer specimens compared with normal breast specimens, and the low TC2N expression was associated with advanced stage, lymphatic metastasis, larger tumors and shorter survival time. Upregulation of TC2N significantly restrains breast cancer cell proliferation in vitro and tumor growth in vivo. Mechanistically, TC2N blocks AKT signaling in a PI3K dependent and independent way through weakening the interaction between ALK and p55γ or inhibiting the binding of EBP1 and AKT. To sum up, these results unmask an ambivalent role of TC2N in cancer, providing a promising inhibitor for PI3K-AKT signaling.

## Introduction

Breast cancer (BC) is a huge growing public health problem and is the most common cancer in women worldwide^1^. Along with the enhancement and development of therapeutic methods and detection strategies, BC patient’s overall survival time is prolonged significantly, with a five-year survival rate of 90%^2^. Despite all this, figuring out the mechanisms underlying the occurrence and development of BC are vital in improving survival of BC patients.

TC2N is a putative C2 domain-containing protein that belongs to the carboxyl-terminal type (C-type) tandem C2 protein family^3^. For years, the role of TC2N in cancer remains completely unexplored. Until recently, we have identified TC2N as a novel oncogene that acts through suppression of the p53 signaling pathway in human lung cancer^4^. Due to the fact that many genes have a dual role in cancer^5,6^, we intend to further explore the precise role of TC2N in cancer development and progression.

In this study, we uncovered a hitherto unknown role of TC2N in BC progression using clinical association analysis, differentially expressed cell models and nude mice. We further showed that TC2N inhibits PI3K-AKT signaling by suppressing phosphorylation of p55γ and AKT. Our findings are the first to demonstrate that TC2N is a new PI3K-AKT signaling suppressor in BC.

## Results

### Elevated expression of TC2N is inversely associated with progression and poor outcome in human BC

**Fig. 1:**
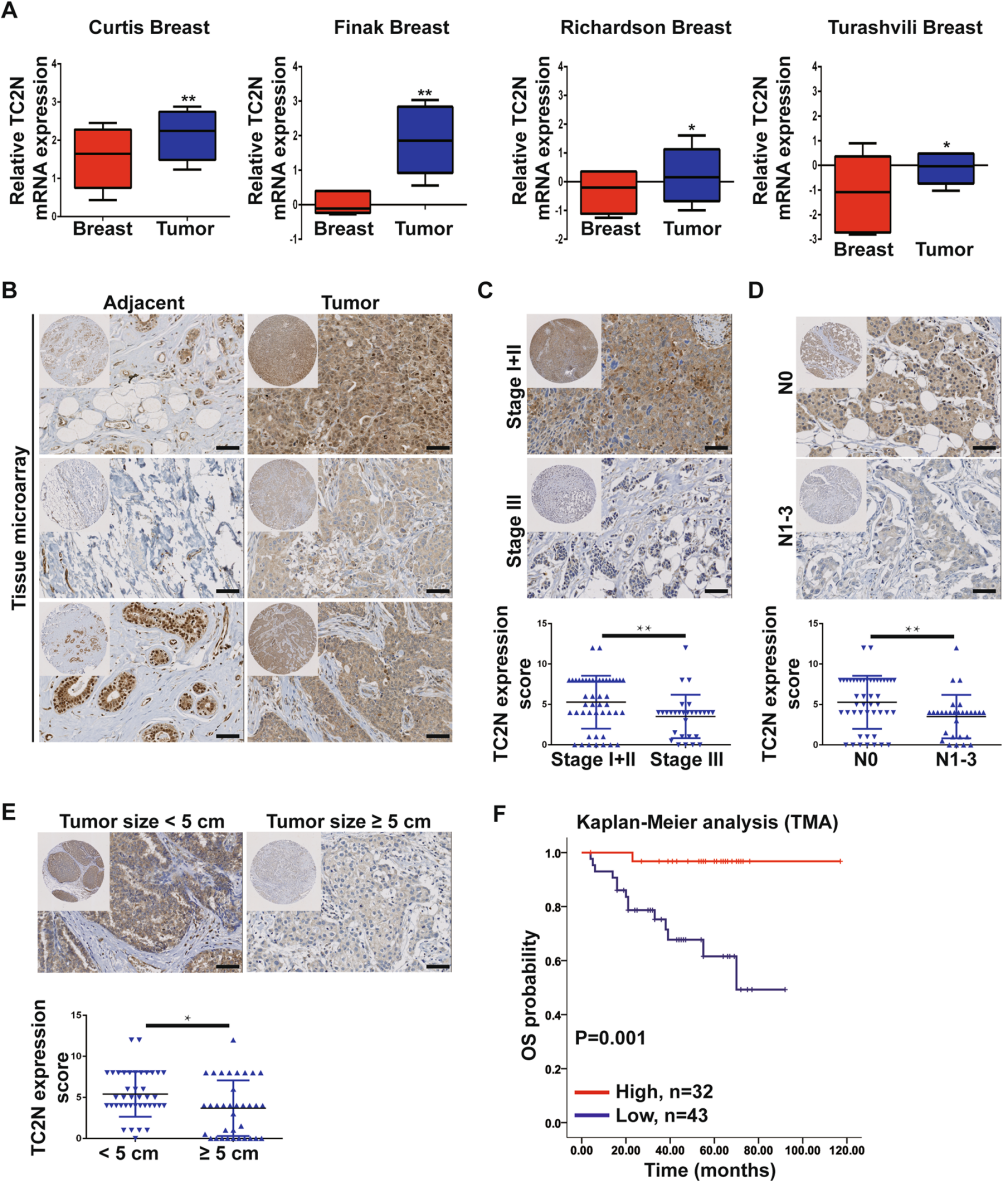
TC2N overexpression in BC predicts better OS and is a valuable prognostic marker of patients with BC. **a** Oncomine BC datasets showing that the mRNA expression levels of TC2N were upregulated in BC tissues. **b** IHC analysis of TC2N protein expression in 75 BC tissues and paired adjacent non-tumor tissues. Scale bars, 20 μm. **c** The protein expression of TC2N was detected in BC patients with different stage of tumor. **d** The protein expression of TC2N was detected in the different stages of BC patients with or without lymphatic metastasis. **e** The protein expression of TC2N was detected in BC patients with different size of tumor. **f** Kaplan–Meier analysis of the correlation between TC2N expression and overall survival time in 75 BC patients. *P < 0.05; **P < 0.01

To investigate the relationship between TC2N and BC, we first assessed the expression of TC2N between tumor and non-tumor tissues by analyzing the published datasets, Oncomine. We found that TC2N mRNA expression was significantly higher in BC tissues than in normal breast specimens (Fig. 1a). These findings were further supported by detecting TC2N protein expression on a tissue microarray containing samples of 75 paired BC specimens and matched normal specimens. Based on IHC results, the protein expression of TC2N was markedly upregulated in tumor tissues compared to adjacent normal tissues non-tumor tissues (Fig. 1b). Furthermore, our statistical analysis revealed a significant association of TC2N protein expression with clinical stage (*P* < 0.001), lymph node metastasis (N) (*P* < 0.001), tumor size (*P* = 0.021) and human epidermal growth factor receptor 2 (HER-2) status (*P* = 0.039), but not with age, histological grade, depth of tumor invasion (T), estrogen receptor (ER) status, progesterone receptor (PR) status and epidermal growth factor receptor (EGFR) status (Fig. 1c–e, Table 1).

**Table 1:** Association of TC2N expression with BC clinicopathological characteristics

Variable	Category	Relative TC2N expression		*P*
		High (*n* = 32)	Low (*n* = 43)	
Age (years)	<45	17	21	0.713
	≥45	15	22	
Clinical stage (AJCC)	I-II	**27**	**19**	<**0.001**
	III	**5**	**24**	
Histological grade	1	2	11	0.091
	2	24	26	
	3	6	6	
Depth of tumor invasion	T_1-2_	18	31	0.154
	_T3-4_	14	12	
Lymph node metastasis	**N**_**0**_	**27**	**19**	<**0.001**
	**N**_**1-3**_	**5**	**24**	
Tumor size	<**5** **cm**	**10**	**25**	**0.021**
	**≥5cm**	**22**	**18**	
PR	Positive	15	23	0.572
	Negative	13	15	
ER	Positive	17	26	0.516
	Negative	11	12	
HER-2	Positive	**27**	**27**	**0.039**
	Negative	**5**	**16**	

Bolded values indicate statistical significance, *P* < 0.05

To further assess the prognostic significance of TC2N in BC, Univariate Kaplan-Meier analysis and multivariate Cox proportional hazards regression analysis were performed to examine the effect of TC2N expression on BC prognosis. As shown in Fig. 1f, Supplementary Fig. S1a and Table 2, high TC2N expression exhibited better outcome of BC patients and was an independent prognostic factor for patient’s overall survival. The same results were obtained by using the online Kaplan Meier plotter tool (Supplementary Fig. S1b). Accordingly, we suspect that TC2N may be a potential tumor suppressor gene in BC.

**Table 2:** Multivariate analysis of different prognostic factors in human BC patients (*n* = 75)

Variables	Multivariate analysis		
	HR	95%CI	*P*
TC2N expression	**0.066**	**0.006–0.680**	**0.022**
Age (years)	2.059	0.551–7.697	0.283
Clinical stage (AJCC)	1.334	0.665–2.678	0.417
Histological grade	2.208	0.752–6.486	0.150
Depth of tumor invasion	1.925	0.350–10.600	0.452
Tumor size	0.626	0.085–4.619	0.626
PR	0.336	0.023–4.917	0.425
ER	1.853	0.122–28.226	0.657

Bolded values indicate statistical significance, *P* < 0.05

*HR* hazard ratio, *CI* confidence interval

### Overexpression of TC2N inhibits breast cancer cell proliferation in vitro and tumor growth in vivo

**Fig. 2:**
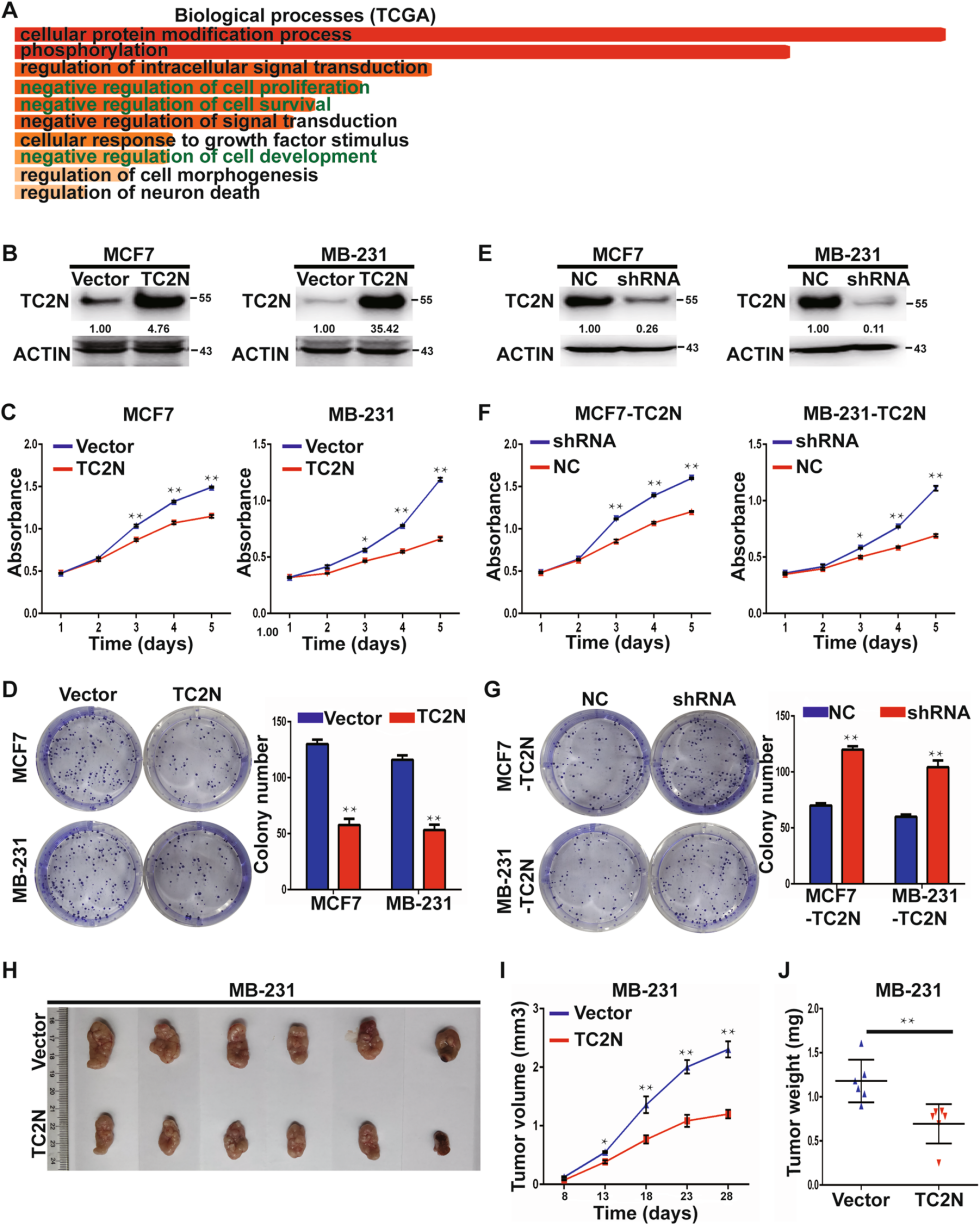
Upregulation of TC2N inhibits BC cell proliferation in vitro and tumor growth in vivo. **a** TCGA BC RNA-seq dataset identified the top 10 categories of the GO biological processes that associate with TC2N expression. **b** MCF7 and MDA-MB-231 cells with TC2N or vector stable transfection were identified by WB. **c** The viability of stable transfected MCF7 and MDA-MB-231 cells were measured by MTS assays. **d** The proliferation of stable transfected MCF7 and MDA-MB-231 cells were measured by colony formation assays. **e** MCF7-TC2N and MDA-MB-231-TC2N cells with NC or shRNA stable transfection were identified by WB. **f** The viability of stable transfected MCF7-TC2N and MDA-MB-231-TC2N cells were measured by MTS assays. **g** The proliferation of stable transfected MCF7-TC2N and MDA-MB-231-TC2N cells were measured by colony formation assays. **h** Photograph of the tumor removed from nude mice at 28 days after inoculated with stable transfected MDA-MB-231 cells. **i** Tumor volume of mice was calculated every 3-5 days. **j** Tumor weights from nude mice were measured. *P < 0.05; **P < 0.01

TC2N expression was inversely correlated with tumor size, which suggested that TC2N may be involved in the regulation of tumor growth. Further Gene ontology (GO) enrichment analysis of a public database, TCGA, showed that co-expressed genes of TC2N were negatively associated with cell proliferation and cell survival (Fig. 2a). To verify the function of TC2N on cell proliferation, we established two TC2N-overexpressing stable BC cell lines by lentiviral transduction (Fig. 2b), and then evaluated the proliferative ability of these cells using MTS and colony formation assays. Indeed, the overexpression of TC2N reduced the viability, colony number and size of BC cells (Fig. 2c, d). In parallel, we knock down TC2N expression in TC2N-overexpressing stable BC cell lines to further confirm the biological functions of TC2N (Fig. 2e). Opposite results were obtained in MTS and colony formation assays, detection of TC2N expression in TC2N-overexpressing stable BC cells resulted in a significant enhancement in proliferation and colony-forming capacity of these cells, revealing the strong anti-tumorigenic function of TC2N (Fig. 2f, g).

Furthermore, the effect of TC2N overexpression on tumorigenesis was examined using nude mice subcutaneous xenograft models. MDA-MB-231-Vector and MDA-MB-231-TC2N cells were subcutaneously injected into the right posterior flanks of nude mice, respectively. The nude mice received TC2N-overexpressing MDA-MB-231 cells formed smaller and lighter tumors than those received vector control cells (Fig. 2h–j).

### Upregulation of TC2N represses PI3K-AKT signaling pathway in breast cancer cells

**Fig. 3:**
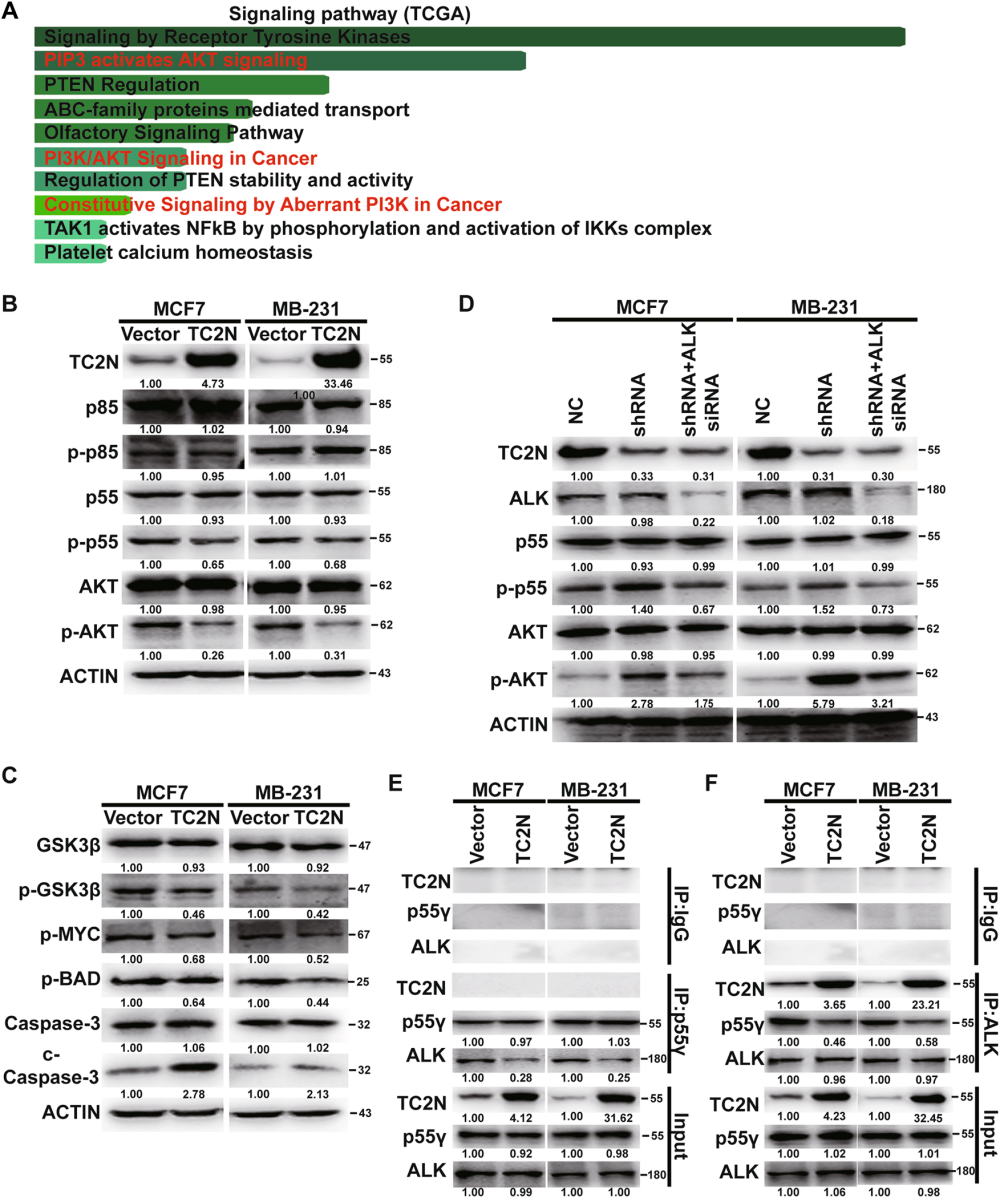
TC2N impedes PI3K-AKT signaling by blocking ALK-induced p55γ phosphorylation in BC cells. **a** TCGA BC RNA-seq dataset identified the top 10 categories of the GO signaling pathway that associate with TC2N expression. **b**, **c** WB analysis of PI3K-AKT signaling-related protein level in stable transfected BC cells. **d** The stable transfected BC cells were transfected with negative control or ALK siRNA for 48 h and then the cells were lysed and were subjected to WB using indicated antibodies. ACTIN serves as an internal control. **e**, **f** The stable transfected BC cell lysates were subjected to IP using p55γ or ALK antibodies and then detected by WB using ALK or p55γ antibodies. Normal IgG serves as a negative control. Whole-cell lysates were used as a positive control (Input)

To uncover the downstream signaling pathway by which TC2N regulates cell proliferation phenotype in BC, we performed GO enrichment analysis using TCGA BC dataset and found that PI3K-AKT signaling pathway was enriched in this dataset (Fig. 3a). Through analysis of the protein expression of PI3K-AKT signaling-related gene, we found that TC2N overexpression did not regulate the phosphorylation level of p85 but instead of decreasing the phosphorylation level of p55γ and AKT (Fig. 3b). Meanwhile, the overexpression of TC2N positively regulates the AKT-suppressed proteins and negatively with AKT-activated proteins (Fig. 3c), indicating that TC2N can inhibit AKT activation.

ALK is a notable activator of PI3K-AKT signaling by specifically inducing the phosphorylation of p55γ subunit of PI3K, rather than more common p85 subunit in cancer^7,8^. As shown in Fig. 3b, the phosphorylation level of p85 remains unchanged after upregulation of TC2N in BC cells. This leads us to suspect whether ALK is involved in TC2N-regulated p55γ dephosphorylation. As expected, the blocking of ALK nullified the inhibitory effect of knockdown of TC2N expression on p55γ and AKT phosphorylation in TC2N-overexpressing cells (Fig. 3d).

Previous studies have confirmed that the interaction between ALK and p55γ is crucial for ALK-induced p55γ phosphorylation^7^. Interestingly, co-IP and WB assays revealed that TC2N could form a complex with ALK, declaring that TC2N might be involved in suppression of ALK binding to p55γ. To clarify this, we conducted competitive co-IP assays in MCF7 and MDA-MB-231 cell lines using ALK or p55γ antibodies. Strikingly, decreased levels of endogenous p55γ protein were detected in ALK precipitates upon TC2N overexpression and vice versa (Fig. 3e, f). Collectively, these results indicated that TC2N attenuates PI3K-AKT signaling through interfering ALK binding to p55γ.

### TC2N restrains PI3K-independent AKT phosphorylation by blocking the interaction of EBP1 with AKT

**Fig. 4:**
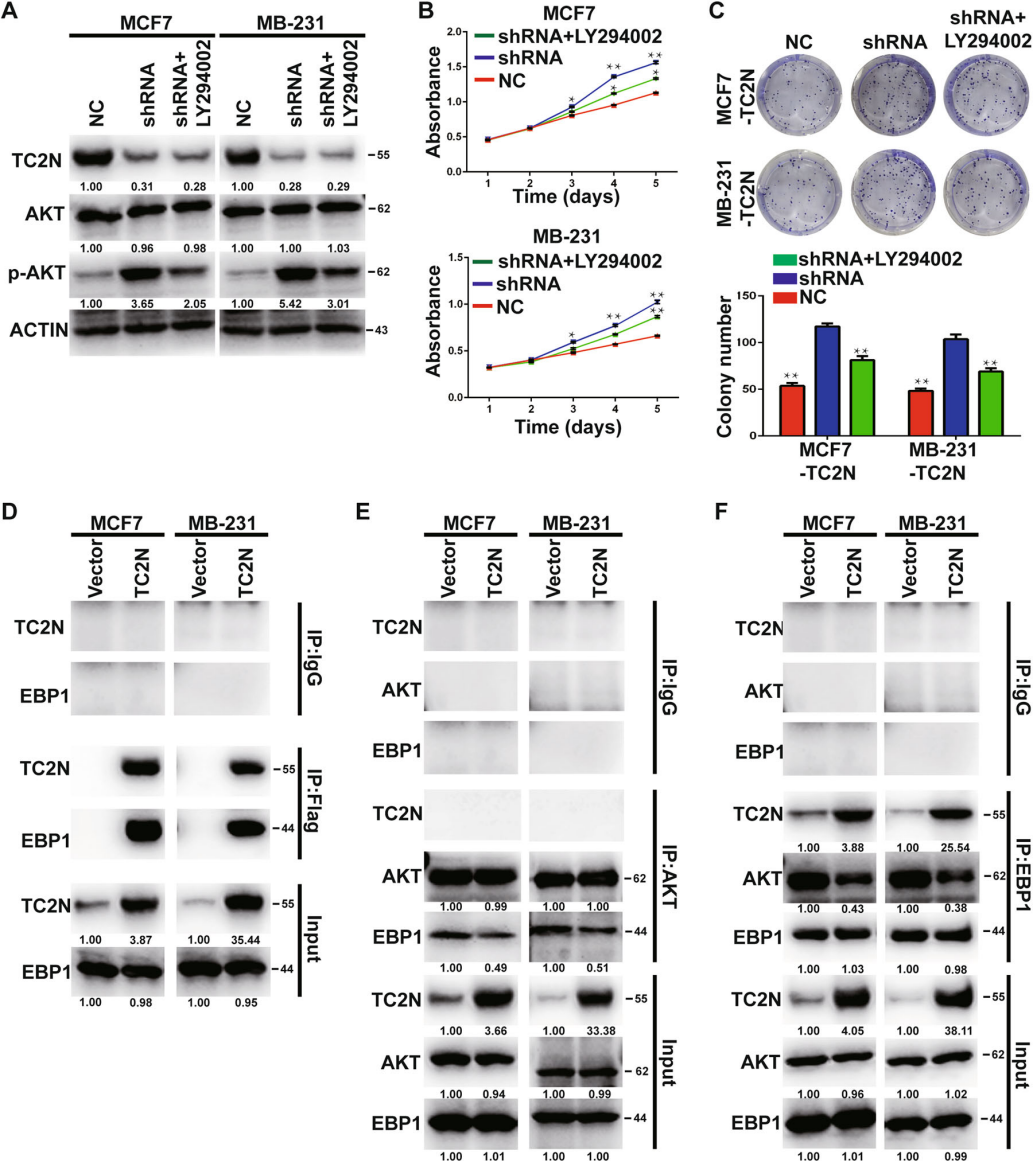
TC2N damages EBP1-induced AKT phosphorylation in BC cells. **a** The protein level of TC2N, AKT and phosphorylated AKT were analyzed by WB after treatment with PI3K inhibitor in stable transfected BC cells. **b** The cell viability was measured by MTS assays after treatment with PI3K inhibitor in stable transfected BC cells. **c** The cell proliferation was measured by MTS assays after treatment with PI3K inhibitor in stable transfected BC cells. **d** The stable transfected BC cell lysates were subjected to IP using Flag antibody and then detected by WB using TC2N and EBP1 antibodies. **e**, **f** The stable transfected BC cell lysates were subjected to IP using AKT or EBP1 antibodies and then detected by WB using EBP1 or AKT antibodies. Normal IgG serves as a negative control. Whole-cell lysates were used as a positive control (Input). *P < 0.05; **P < 0.01

Next, we investigated whether PI3K activation was required for the ability of TC2N to inhibit cell proliferation in BC cells. WB results revealed that only part of the phosphorylation level of AKT TC2N-induceddephosphorylation of AKT was rescued by LY294002, a known inhibitor of PI3K when TC2N knockdown (Fig. 4a). Correspondingly, we observed that the inhibitory effect of TC2N overexpression on cell proliferation was partly restored (Fig. 4b, c), accompanied by PI3K suppression, suggesting that TC2N-mediated dephosphorylation of AKT may involve in a PI3K-independent pathway.

To test this hypothesis, we plan to identify the TC2N-interacting proteins which are associated with AKT phosphorylation. TC2N-overexpressing MCF7 and MDA-MB-231 cell lysates were immunoprecipitated with Flag antibody and then the immunoprecipitate was subjected to further proteomic analysis. As shown in Table S2, a total of 282 and 246 proteins were identified in MCF7-TC2N and MDA-MB-231-TC2N cells, respectively. Among these proteins, EBP1, one of which can accelerate AKT phosphorylation, that capture our attention. Previous studies demonstrated that EBP1 controls the phosphorylation level of AKT depends on interaction of EBP1 with AKT^9,10^. Encouraged by our above results, we next examined whether TC2N can attenuate AKT phosphorylation via competing to bind EBP1 with AKT. To validate this hypothesis, we performed co-IP assays in MCF7 and MDA-MB-231 cells and found that EBP1 can be co-immunoprecipitated with TC2N in MCF7 and MDA-MB-231 cells (Fig. 4d). Further, the result of competitive co-IP assays revealed that TC2N can recede the interaction of EBP1 with AKT (Fig. 4e, f).

### Blocking of PI3K-AKT signaling leads to attenuate the suppression effect of TC2N overexpression on cell proliferation in BC cells

**Fig. 5:**
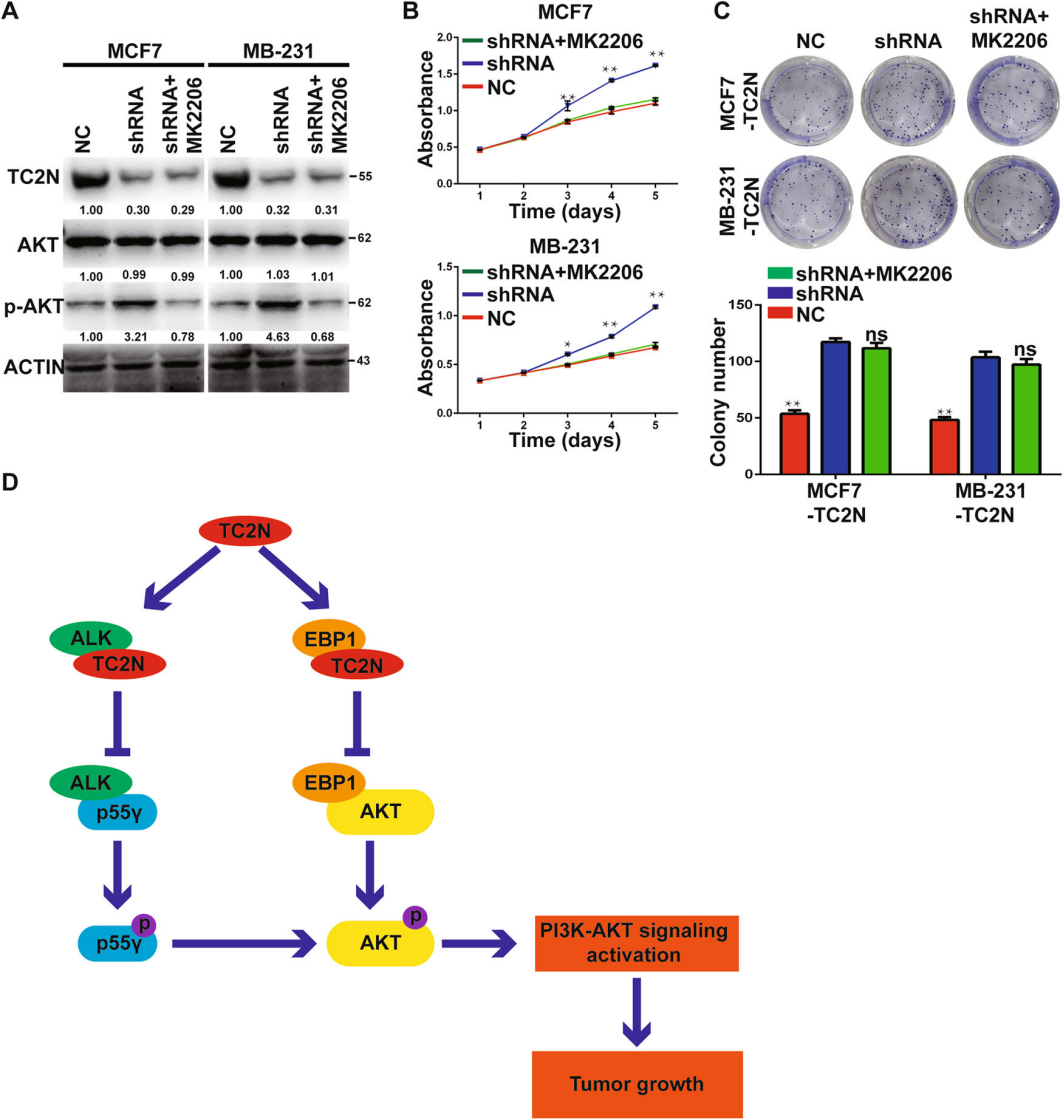
PI3K-AKT signaling is indispensable for the anti-tumor function of TC2N. **a** The protein level of TC2N, AKT and phosphorylated AKT were analyzed by WB after treatment with AKT inhibitor in stable transfected BC cells. The cell viability was measured by MTS assays after treatment with AKT inhibitor in stable transfected BC cells. **c** The cell proliferation was measured by MTS assays after treatment with AKT inhibitor in stable transfected BC cells. **d** A schematic illustration of how TC2N suppresses tumor progression in BC. *P < 0.05; **P < 0.01

To further determine whether PI3K-AKT signaling is a critical mediator of TC2N in proliferation of BC cells, the activation of PI3K-AKT signaling was blocked by PI3K inhibitor and AKT inhibitor in TC2N-overexpressing BC cells when TC2N was silenced (Fig. 5a). Indeed, suppression of PI3K-AKT signaling strongly reversed the stimulatory effect of TC2N knockdown on cell proliferation (Fig. 5b, c).

## Discussion

In previous studies, we have demonstrated that the expression of TC2N is upregulated in lung tumor tissues compare with the paired adjacent non-cancerous tissues from the same patient^4^. Upregulated TC2N promoted tumor growth and metastasis and is associated with high histological grade, advanced clinical stage, and thus a poor clinical prognosis of lung cancer patients^4^. In our present study, we found that TC2N is also frequently overexpressed in BC tissues, but is under expressed in adjacent normal tissues, which is coordinated with the expression pattern of TC2N in lung cancer. So, does that mean TC2N is still an oncogene in BC? Oppositely, high TC2N expression predicts favorable prognosis in BC, suggesting that TC2N may be a tumor suppressor in BC.

Clinically, depending on the expression status of estrogen receptor (ER), progesterone receptor (PR) and ERBB2 receptor (HER-2), BC can be classified into four major subtypes including luminal A (ER positive and/or PR positive, HER-2 negative), luminal B (ER positive and/or PR positive, HER-2 positive), HER2 (ER negative, PR negative, HER-2 positive), basal-like (ER negative, PR negative, HER-2 negative)^11,12^. And while these four subtypes belong to BC, their incidence, risk factors, prognosis and therapeutic strategy are different^13^. For that reason, a clear understanding of the differences of molecular characteristics of these four subtypes of BC are vital in leading to deeper understanding and administration of targeted therapeutics. In this study, we found that TC2N expression is associated with HER-2 expression (P = 0.039), but there is no relation between TC2N expression and the expression of ER and PR (Table 1). However, a surprising finding was evidence of the expression of TC2N in ER high expression group, PR high expression group and HER-2 high expression group were higher than in ER low expression group, PR low expression group and HER-2 low expression group through analysis of TCGA and Oncomine database (Supplementary Fig. S2). There are two possible explanations for these findings: 1) TC2N expression is indeed associated with triple-negative breast cancer (TNBC), but the small sample size of TMA limit our analysis. 2) TC2N expression is only associated with HER-2 positive breast cancer. These means that the clinical significance of TC2N in BC should be further replicated and explored with a larger sample.

Genes with similar expression patterns always share similar functions^14,15^. In this study, the co-expressed genes of TC2N were enriched in cell proliferation, cell survival and cell development signatures, which hint that TC2N may be participated in regulation of cell proliferation. Consistently, upregulation of TC2N dramatically reduced, whereas knockdown of TC2N rescued, the cell proliferation in vitro and tumor growth in vivo. These data uncover an ambivalent role of TC2N in cancer. However, the downstream signaling pathway and molecular mechanisms underlying the tumor suppressive effect of TC2N remains obscure. To gain insight into this issue, we tried to investigate the possible downstream signaling, and found that the different effects on tumor growth of ectopic expression of TC2N are associated with PI3K-AKT signaling.

Hyperactivation of PI3K-AKT signaling pathway is considered as a hallmark in a wide spectrum of human cancers^16–18^. The most of studies have demonstrated that PI3K-AKT signaling involves in regulation of metabolism, growth, survival, angiogenesis and metastasis of tumor cell^19,20^. Activation of PI3K phosphorylates (catalyzes) phosphatidylinositol-4,5-bisphosphate (PIP2) to phosphatidylinositol-3,4,5-trisphosphate (PIP3), which acts as a second messenger to recruit a subset of signaling proteins that contain pleckstrin homology (PH) domains to the membrane, including AKT and phosphoinositide dependent kinases (PDKs) 1/2. Then, PDKs phosphorylate and activate AKT. Activated AKT moves to the cytoplasm and nucleus, resulting in the activation of a host of downstream targets that control cell proliferation, cell cycle progression, cell survival, angiogenesis and metastasis^21^. Therefore, blocking of PI3K or AKT may provide an effective therapeutic approach for cancer. Although numerous inhibitors of PI3K and AKT have been identified, these drugs are still too long to be applied to clinical practice because single agents tends to develop drug resistance^22,23^.

In our research, TC2N declines the phosphorylation level of PI3K by competing with p55γ for ALK binding. Besides, we also noticed that TC2N can facilitate the dephosphorylation of PTEN (Supplementary Fig. S3), which acts as a critical negative regulator of PI3K functionality^24,25^. This means that, TC2N might prevent the transition from PIP3 to PIP2 via activation of PTEN. Then, it deserves to be further investigated. Furthermore, TC2N could hinder AKT phosphorylation by competing with AKT for EBP1 binding. These data suggest that TC2N is a robust repressor of PI3K-AKT signaling pathway in BC.

## Conclusion

In summary, we here provide evidence that high expression of TC2N inhibits cell proliferation and tumor growth and correlates with a better prognosis in BC. Mechanistically, TC2N suppresses PI3K-AKT signaling through blocking p55γ and AKT phosphorylation. More importantly, our studies suggest that TC2N may serve as a valuable prognosis indicators and is a promising inhibitor of PI3K-AKT signaling for BC. Of course, more basic and clinical studies are required to further elucidate the association between TC2N and PI3K-AKT signaling in BC.

## Materials and methods

### Cell lines

The BC cell lines (MCF7 and MDA-MB-231) were purchased from the Cell Bank of the Chinese Academy of Science (Shanghai, China). Cells were cultured in DMEM mediums (Gibco, CA) supplemented with 10% fetal bovine serum (Gibco, CA) and maintained at 37 °C with 5% CO_2_.

### Tissue microarray (TMA) and immunohistochemical (IHC) analysis

TMA contained 75 breast tumor samples and 75 adjacent noncancerous breast tissues were produced by the collaboration (Shanghai Biochip Co Ltd, Shanghai, People’s Republic of China). IHC staining was performed using a rabbit monoclonal antibody against TC2N (1:500; Abcam) as described previously^4^. The quantitative methods for evaluating protein expression of TC2N are described in a previous study^26^. The study was approved by the Ethics Committee of Third Military Medical University and all patients signed the written informed consent. The clinical and pathological features of these patients are described in Supplementary Table S1.

### Plasmid construction, retroviral infection and cell transfection

For overexpression, the full-length open reading frame of human TC2N was generated by synthesis and subsequent molecular cloning into pMSCV retrovirus plasmid. The TC2N lentivirus was constructed and packaged by Genomeditech (Shanghai, China). For knockdown, a hairpin precursors presented high efficiency in knocking down TC2N was constructed as previously described^4^. Cells were transfected using Lipofectamine2000 Reagent (Invitrogen Preservation, Carlsbad, CA, USA) according to the manufacturer’s instructions. The stably transfected cells were screened under Puromycin (Sigma).

### Cell proliferation assay

The stable transfected MCF7 and MDA-MB-231 cells were plated in 96-well plates at a density of 3000 cells/well. On day 1–5, MTS (promega, USA) reagent was added and incubated at 37 °C for 1 h, and the absorbance values of each well were measured at 490 nm using a spectrophotometer.

### Colony formation assay

For colony formation assay, 200 stable transfected cells were resuspended and seeded into 6-well plates and cultured with DMEM containing 10% FBS at 37 °C. After incubation for two weeks, colonies were fixed in 4% paraformaldehyde for 20 min and stained with 0.1% crystal violet for 5 min. Cell colonies which contain more than 50 cells were then counted.

### WB analysis

WB was performed as previously described^27^. The following primary antibodies were used: TC2N rabbit polyclonal antibody (1:500; Abcam), p85 mouse monoclonal antibody (1:1000; Santa Cruz), p-p85 Tyr508 goat polyclonal antibody (1:1000; Santa Cruz), p-p55γ Tyr199 rabbit polyclonal antibody (1:1000; Bioss), p55γ mouse monoclonal antibody (1:1000; Santa Cruz), AKT mouse monoclonal antibody (1:1000; Santa Cruz), p-AKT Ser473 mouse monoclonal antibody (1:1000; Santa Cruz) GSK3B mouse monoclonal antibody (1:1000; Santa Cruz), p-GSK3B mouse monoclonal antibody (1:1000; Santa Cruz), p-MYC mouse monoclonal antibody (1:1000; Santa Cruz), p-BAD mouse monoclonal antibody (1:1000; Santa Cruz), Caspase-3 mouse monoclonal antibody (1:1000; Santa Cruz), cleaved-Caspase-3 rabbit monoclonal antibody (1:1000; Cell Signaling Technology), ALK mouse monoclonal antibody (1:1000; Santa Cruz), EBP1 mouse monoclonal antibody (1:1000; Santa Cruz) and ACTIN monoclonal antibody (1:2000; Sigma). ACTIN served as a loading control.

### Co-Immunoprecipitation (Co-IP) assay and iTRAQ-labeling proteomic analysis

Total extracts of MCF7 and MB-231 with or without TC2N overexpression were lysed with IP lysis buffer (Beyotime, China). The co-IP analyses were performed using a Co-Immunoprecipitation Kit (Pierce, US) according to the manufacturer’s protocol. Subsequent WB analyses were performed as described above. The experiment was repeated thrice.

For proteomic analysis, the cell lysates were further analyzed by using a NanoLC system (NanoLC-2D Ultra, Eksigent) as previously described^28^. Based on the Swiss-Prot Homo sapiens protein databases, which were released in November 2012 (84,736 proteins) and May 2013 (88,631 proteins), the acquired proteins were identified by using ProteinPilot 4.5 software (AB SCIEX, USA). All of these proteins are listed in Table S2.

### Xenograft tumor growth model

For in vivo tumor growth experiment, a total of 5 × 10^6^ stable transfected cells suspended in 200 μl PBS were injected into the right flanks of the nude mice. Tumors size was measured every 3–5 days with vernier caliper after injection, and the tumor volume was calculated based on formula: 0.5 × (length × width^2^). After 28 days housing, mice were euthanized, tumors were excised and weighed. The images of tumor were taken. All experiments on mice were approved by the Institutional Animal Care and Use Committee of Third Military Medical University, China.

### Gene ontology (GO) analysis and Kaplan Meier plotter

For GO analysis, the RNA-Seq data from TCGA database was used to analyze the correlation between TC2N expression and all other genes in patients with BC. All 13949 genes (Data not shown) significantly correlated with TC2N expression were used for GO analysis by using the Gene Ontology Consortium tool (http://www.geneontology.org/).

The online Kaplan Meier plotter tool were used to verify the prognostic value of TC2N as previously described^29^.

### Statistical analysis

Statistical analyses were performed with the SPSS 16.0 software (SPSS, Inc., Chicago, IL, USA). Each experiment was performed at least three times. The data were presented as the means ± SD. The statistical comparisons were analyzed using Chi-square test, Student’s t-test (only two groups) or One-way ANOVA (three or four groups). The correlation between the expression of TC2N and the clinical pathologic feature of BC patients was analyzed by the Pearson’s chi-squared test or Fisher’s exact test. The Kaplan Meier method was used to assess the relationship between TC2N and OS in BC patients. Cox regression models were used to analyze independent prognostic factors. Correlation analysis of gene expression was performed using Spearman’s rank correlation coefficient analysis. A two-sided *P*-value <0.05 was taken as statistically significant.

## Supplementary information

Figure S1

Figure S2

Figure S3

Table S1

Table S2

supplement information
