# Author Correction: Identification of TC2N as a novel promising suppressor of PI3K-AKT signaling in breast cancer

**DOI:** 10.1038/s41419-019-1726-7

**Published:** 2019-06-24

**Authors:** Xiang-lin Hao, Li-yun Gao, Xiao-juan Deng, Fei Han, Hong-qiang Chen, Xiao Jiang, Wen-bin Liu, Dan-dan Wang, Jian-ping Chen, Zhi-hong Cui, Lin Ao, Jia Cao, Jin-yi Liu

**Affiliations:** 1 Institute of Toxicology, College of Preventive Medicine, Third Military Medical University, Chongqing, 400038 PR China; 2 School of Public Health, Xinxiang Medical University, Xinxiang, PR China; 3 Cooperative innovation center of molecular diagnosis and medical inspection technology, Beijing, PR China

**Keywords:** Breast cancer, Phosphorylation

**Correction to: Cell Death & Disease**

10.1038/s41419-019-1663-5 Published online 29 May 2019

After publication of this article, it came to the attention of the authors that their names had been reordered. Professor. Jia Cao and Prof. Jin-yi Liu are the co-corresponding authors, and Prof. Jin-yi Liu should be the last author.

This has been corrected in both the PDF and HTML versions of the Article.

