# Supplementary figures and images for: Identification of TC2N as a novel promising suppressor of PI3K-AKT signaling in breast cancer
Source: Cell Death Dis. 2019 May 29;10(6):424. doi: 10.1038/s41419-019-1663-5 (PMC6541591; doi:10.1038/s41419-019-1663-5)

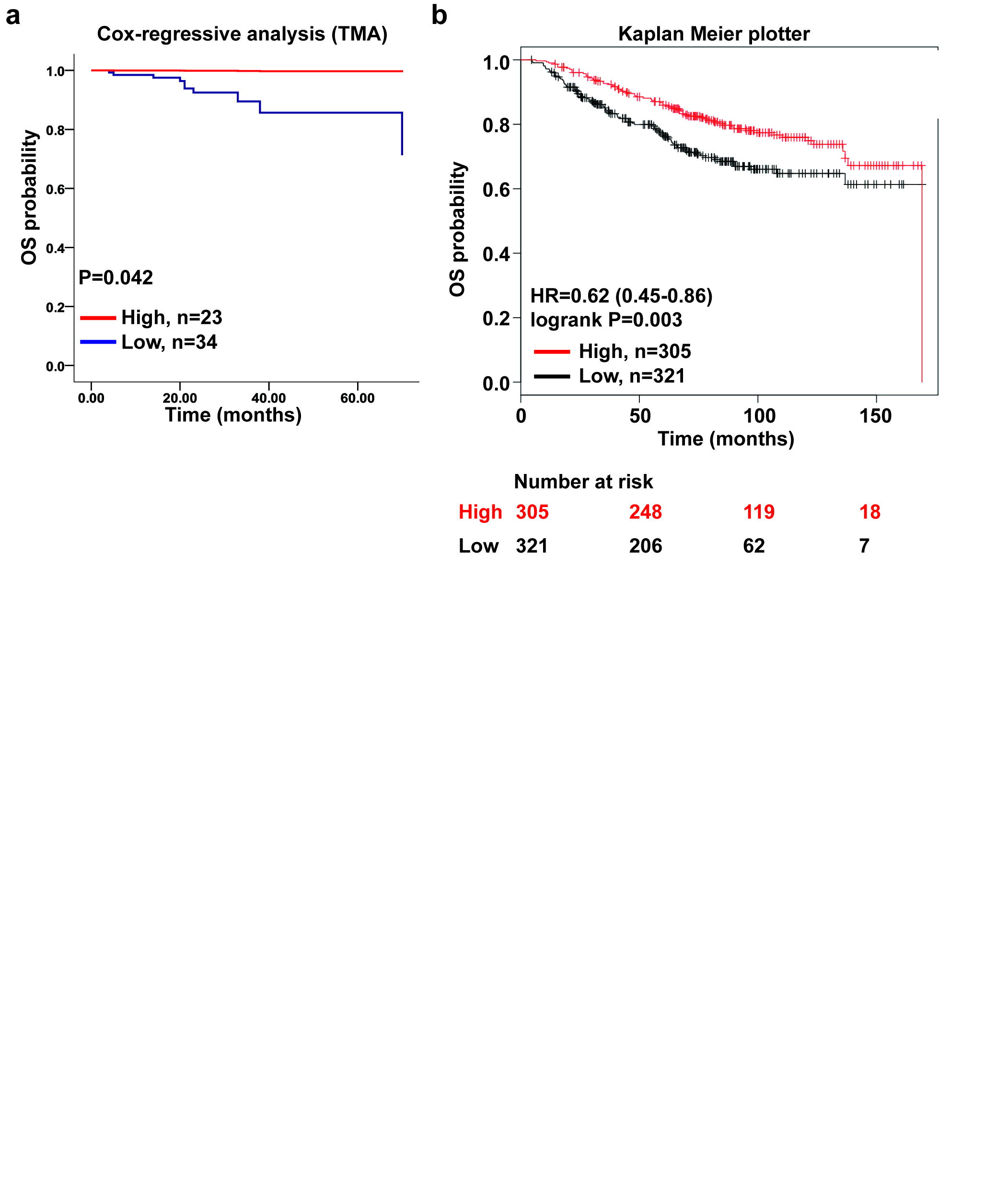

Supplement: Supplementary file 1 — Figure S1 [file 41419_2019_1663_MOESM1_ESM.tif]

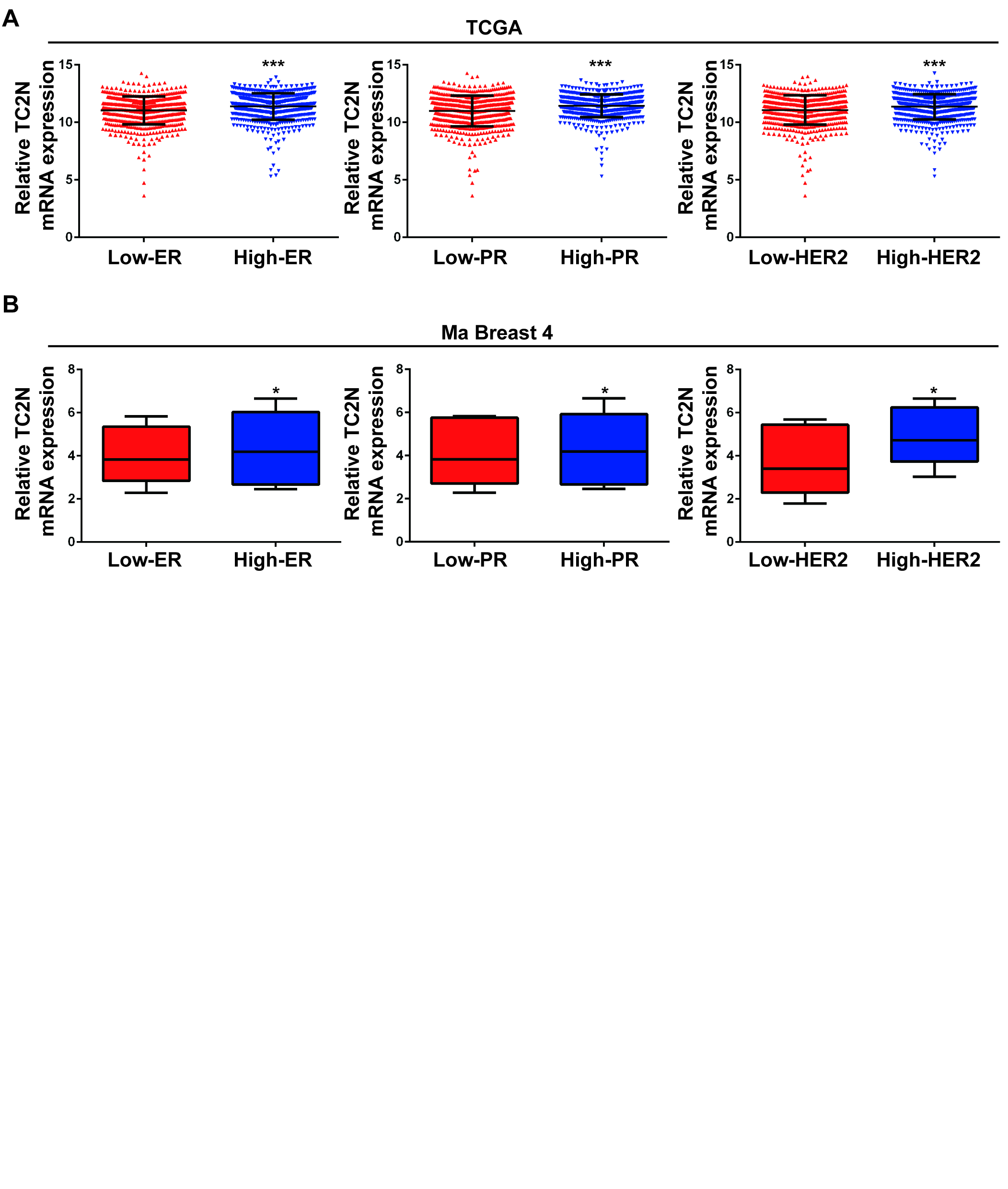

Supplement: Supplementary file 2 — Figure S2 [file 41419_2019_1663_MOESM2_ESM.tif]

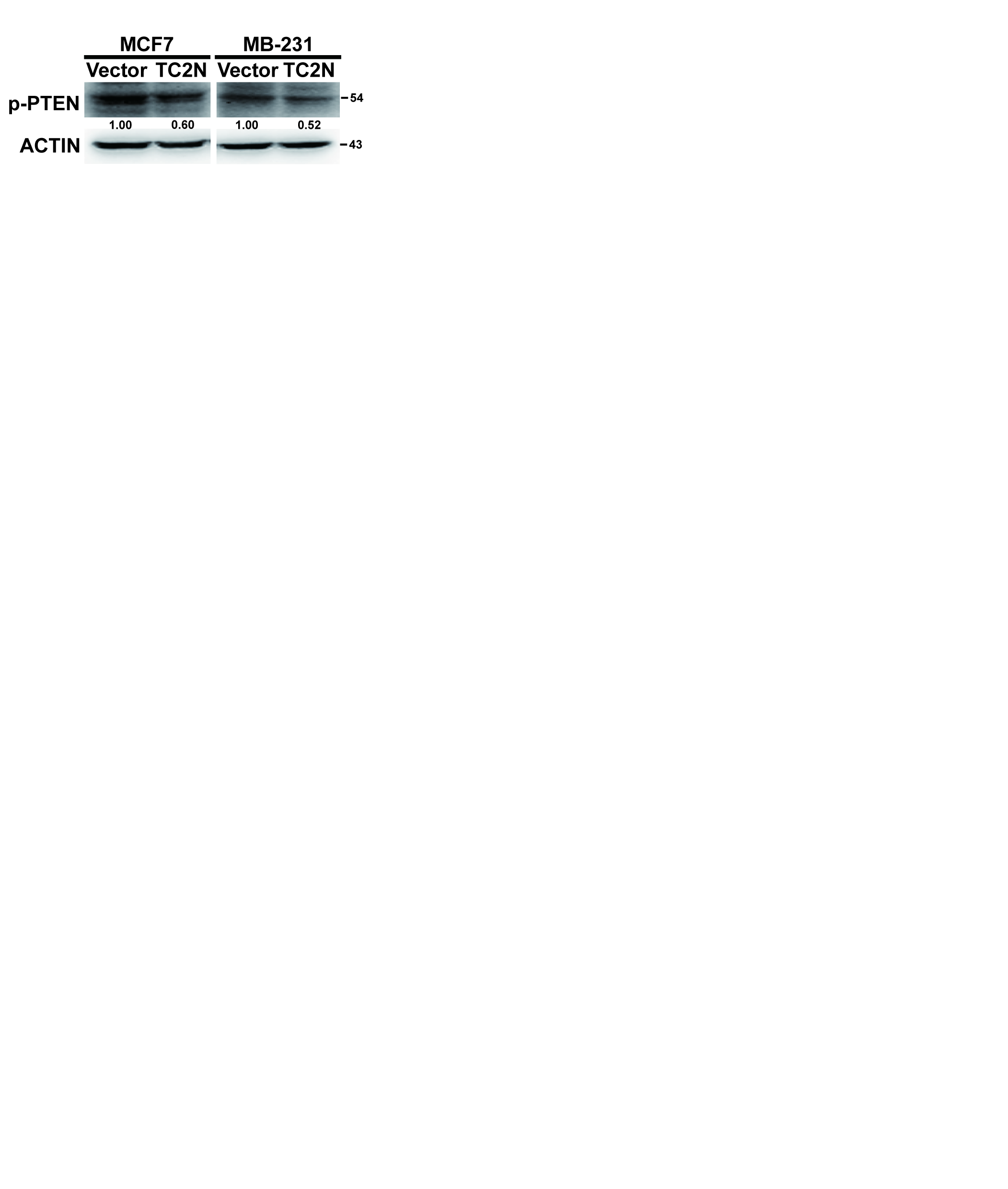

Supplement: Supplementary file 3 — Figure S3 [file 41419_2019_1663_MOESM3_ESM.tif]
