# Supplementary material for: Identification of TC2N as a novel promising suppressor of PI3K-AKT signaling in breast cancer
Source: Cell Death Dis. 2019 May 29;10(6):424. doi: 10.1038/s41419-019-1663-5 (PMC6541591; doi:10.1038/s41419-019-1663-5)
Supplement: Supplementary file 4 — Table S1 [file 41419_2019_1663_MOESM4_ESM.docx]

|  | | |
| --- | --- | --- |
| **Characteristic** |  | **Number of Patients** |
| Patients |  | 75 |
| Age (years) |  | 27-80, median=44 |
| Clinical stage (AJCC) |  |  |
| Ⅰ |  | 30 |
| Ⅱ |  | 16 |
| Ⅲ |  | 29 |
| Histological grade |  |  |
| 1 |  | 13 |
| 2 |  | 50 |
| 3 |  | 12 |
| Depth of tumor invasion |  |  |
| T_1_ |  | 1 |
| T_2_ |  | 48 |
| T_3_ |  | 25 |
| T_4_ |  | 1 |
| lymph node metastasis |  |  |
| N_0_ |  | 46 |
| N_1_ |  | 25 |
| N_2_ |  | 4 |
| N_3_ |  | 0 |
| Tumor size (cm) |  | 1.0-17.5, median=3.0 |
| PR status |  |  |
| Positive |  | 38 |
| Negative |  | 28 |
| ER status |  |  |
| Positive |  | 43 |
| Negative |  | 23 |
| HER-2 status |  |  |
| Positive |  | 54 |
| Negative |  | 21 |
| Clinical stage, tumor-nodes-metastasis, based on the American Joint Committee on Cancer/International Union Against Cancer Staging Manual (7th edition, 2009) | | |
|  |  |  |
|  |  |  |
