# Supplementary material for: Identification of TC2N as a novel promising suppressor of PI3K-AKT signaling in breast cancer
Source: Cell Death Dis. 2019 May 29;10(6):424. doi: 10.1038/s41419-019-1663-5 (PMC6541591; doi:10.1038/s41419-019-1663-5)
Supplement: Supplementary file 6 — supplement information [file 41419_2019_1663_MOESM6_ESM.docx]

**Supplementary Figure legends**

**Supplementary Fig. S1.** High TC2N expression is correlated with longer survival time in BC patients. A: Cox regression survival analysis of TC2N expression in 75 BC patients split into two groups. B: Kaplan-Meier survival analysis of TC2N expression with OS by using Kaplan Meier plotter.

**Supplementary Fig. S2.** Analysis of TC2N expression in different subtypes of BC. A: The expression of TC2N in different subtypes of BC were analyzed using TCGA database. B: The expression of TC2N in different subtypes of BC were analyzed using Oncomine database.

**Supplementary Fig. S3.** The phosphorylation level of PTEN was monitored by WB after overexpression of TC2N in BC cells. ACTIN serves as an internal control.

**Supplementary Tables**

**Supplementary Table 1.** Clinicopathologic characteristics of patients.

**Supplementary Table 2.** The proteins co-precipitates with TC2N.
